# EDC IMPACT: Reduced sperm counts in rats exposed to human relevant mixtures of endocrine disrupters

**DOI:** 10.1530/EC-17-0307

**Published:** 2017-11-28

**Authors:** M Axelstad, U Hass, M Scholze, S Christiansen, A Kortenkamp, J Boberg

**Affiliations:** 1 Technical University of DenmarkNational Food Institute, Kongens Lyngby, Denmark; 2 Brunel UniversityUxbridge, UK

**Keywords:** semen quality, endocrine disrupters, acetaminophen, phthalates, pesticides, estrogenic, anti-androgenic

## Abstract

Human semen quality is declining in many parts of the world, but the causes are ill defined. In rodents, impaired sperm production can be seen with early life exposure to certain endocrine-disrupting chemicals, but the effects of combined exposures are not properly investigated. In this study, we examined the effects of early exposure to the painkiller paracetamol and mixtures of human relevant endocrine-disrupting chemicals in rats. One mixture contained four estrogenic compounds; another contained eight anti-androgenic environmental chemicals and a third mixture contained estrogens, anti-androgens and paracetamol. All exposures were administered by oral gavage to time-mated Wistar dams rats (*n* = 16–20) throughout gestation and lactation. In the postnatal period, testicular histology was affected by the total mixture, and at the end of weaning, male testis weights were significantly increased by paracetamol and the high doses of the total and the anti-androgenic mixture, compared to controls. In all dose groups, epididymal sperm counts were reduced several months after end of exposure, i.e. at 10 months of age. Interestingly, the same pattern of effects was seen for paracetamol as for mixtures with diverse modes of action. Reduced sperm count was seen at a dose level reflecting human therapeutic exposure to paracetamol. Environmental chemical mixtures affected sperm count at the lowest mixture dose indicating an insufficient margin of safety for the most exposed humans. This causes concern for exposure of pregnant women to paracetamol as well as environmental endocrine disrupters.

## Introduction

Human semen quality is declining in many parts of the world, and this has recently been confirmed by a comprehensive meta-analysis showing declining sperm counts in North America, Europe, Australia and New Zealand over the last four decades (1). The causes of this decline are ill-defined, and environmental influences including chemical exposures have been suggested (2). This is supported by rodent models showing impaired sperm production after early life exposure to certain endocrine-disrupting chemicals (EDCs).

Numerous EDCs are present in our environment, and EDCs are suspected of playing a role in testicular dysgenesis syndrome (TDS) in humans. This syndrome encompasses a range of male disorders, presumed to have common developmental origins: hypospadias, cryptorchidism and short anogenital distance in boys, and low sperm quality and high testis cancer risk in adult men (3). In the adult organism, functional hormonal feedback regulation ensures that appropriate homeostasis is obtained, but during sexual development, exposure to certain chemicals can disturb this hormonal balance, and even short-term exposures can cause adverse and permanent changes to the reproductive system.

The present study aimed at investigating how male reproduction may be influenced by developmental exposure of rats to the painkiller paracetamol or to mixtures of human relevant environmental EDCs. In addition to a therapeutically relevant dose of paracetamol, a mixture of 8 anti-androgenic EDCs, another mixture of 4 estrogenic EDCs and a total mixture of all 12 EDCs and paracetamol were investigated. The mixture composition for the environmental EDCs was based on high-end human intakes, as described in detail by Christiansen and coworkers (4). The 13 compounds were selected as representative of the numerous other EDCs that humans are exposed to, and the mixture ratio was intended to reflect a realistic human intake.

The 8 environmental chemicals with anti-androgenic mode of action were the two phthalates, di-n-butyl phthalate (DBP), di-(2-ethylhexyl) phthalate (DEHP), the five pesticides vinclozolin, prochloraz, procymidone, linuron, epoxiconazole and the pesticide metabolite dichlorodiphenyl-dichloroethylene (p,p′-DDE). The four predominantly estrogenic substances were the two UV-filters octyl methoxycinnamate (OMC) and 4-methyl-benzylidene camphor (4-MBC), the phenolic compound bisphenol A (BPA) and the preservative butyl paraben. The effects of each subgroup were compared to the effects of the total mixture to examine the contribution of each subgroup to the overall effects. For some of the chemicals, we made adjustments to reflect the fact that only certain population groups are highly exposed, and selected compounds were used as a proxy for a group of related chemicals; e.g. DBP and DEHP also representing other known anti-androgenic phthalates and selected pesticides representing a larger group of other anti-androgenic pesticides.

In other papers on this study, we have described reduced male anogenital distance, increased nipple retention, decreased weights of ventral prostate and of the levator ani/bulbocavernosus muscle (LABC) in prepuberty (5), and adverse changes in prostate and mammary gland development were seen in these animals in puberty and adulthood (6, 7, 8) (Supplementary Table 1, see section on supplementary data given at the end of this article). The current paper focuses on long-term adverse effects on sperm count in the offspring.

## Materials and methods

### Test compounds

The test compounds were DBP (purity >99.0%, CAS no. 84-74-2), DEHP (purity >99.5%, CAS no. 117-81-7), vinclozolin (purity >99.5%, CAS no. 50471-44-8), prochloraz (purity >98.5%, CAS no. 67747-09-5), procymidone (purity >99.5%, CAS no. 32809-16-8), linuron (purity >99.0%, CAS no. 330-55-2), epoxiconazole (purity >99.0%, CAS no. 106325-08-8), OMC (purity >98.0%, CAS no. 5466-77-3), p,p′-DDE (purity >98.5%, CAS no.72-55-9), which were all purchased from VWR – Bie & Berntsen (Herlev, Denmark). While 4-MBC (purity >98.0%, CAS no. 36861-47-9), bisphenol A (purity >99.5%, CAS no. 80-05-7), butyl paraben (purity >99.0%, CAS no. 94-26-8) and paracetamol (purity >99.0%, CAS no. 103-90-2), were purchased from Sigma-Aldrich. Corn oil used both as a control compound and as a vehicle was purchased from VWR – Bie & Berntsen.

### Animals and exposure

The animal experiment was carried out at the DTU National Food Institute (Mørkhøj, Denmark) facilities. Ethical approval was given by the Danish Animal Experiments Inspectorate. The authorization number was 2012-15-2934-00089 C4. The experiments were overseen by the National Food Institutes in-house Animal Welfare Committee for animal care and use. Detailed description on animal housing and test substance exposure is presented by Axelstad and coworkers (5). In summary, 156 time-mated nulliparous, young adult Wistar rats (HanTac:WH) were supplied at gestation day (GD) 3 of pregnancy. The day when a vaginal plug was detectable was designated as gestation day (GD) 1 and independently of actual day of delivery, the expected day of delivery, GD 23 was designated as pup day (PD) 1. Thereby, the age of the pups related to the time of conception, but was rather similar to postnatal age. The animals were housed in pairs until GD 17 and alone thereafter under standard conditions in polycarbonate cages. They were placed in an animal room with controlled environmental conditions, fed a standard soy- and alfalfa-free diet and received acidified tap water *ad libitum* (see 5 for details on housing conditions).

On the day after arrival (GD 4), the time-mated dams were pseudo-randomly distributed into 9 groups with similar body weight (bw) distributions. Between 16 and 20 mated dams were included in each dose group, resulting in 14–20 viable litters per group. The dams received vehicle (controls) or one of the eight mixtures. These included 3 doses of total mixture (TotalMix100, TotalMix200, TotalMix450), 2 doses of anti-androgens only (AAMix200, AAMix450), 2 doses of estrogens only (EMix200, EMix450) and paracetamol (PM). Dams that did not give birth were omitted from the experiment. Details on mixture composition and doses of each compound are shown in Table 1.

**Table 1:** Composition of the tested mixtures.

		**Mixture dose** (mg/kg day)							
**Chemical**	Adjusted human intakes chosen as basis for the mixture study* (mg/kg day)	TotalMix 100	TotalMix 200	TotalMix 450	AAMix 200	AAMix 450	EMix 200	EMix 450	Paracetamol
DBP	0.01	1	2	4.5	2	4.5	0	0	0
DEHP	0.02	2	4	9	4	9	0	0	0
Vinclozolin	0.009	0.9	1.8	4.05	1.8	4.05	0	0	0
Prochloraz	0.014	1.4	2.8	6.3	2.8	6.3	0	0	0
Procymidone	0.015	1.5	3	6.75	3	6.75	0	0	0
Linuron	0.0006	0.06	0.12	0.27	0.12	0.27	0	0	0
Epoxiconazole	0.01	1	2	4.5	2	4.5	0	0	0
p,p′-DDE	0.001	0.1	0.2	0.45	0.2	0.45	0	0	0
4-MBC	0.06	6	12	27	0	0	12	27	0
OMC	0.12	12	24	54	0	0	24	54	0
Bisphenol A	0.0015	0.15	0.3	0.675	0	0	0.3	0.675	0
Butyl paraben	0.06	6	12	27	0	0	12	27	0
Paracetamol^a^	0.8	80	160	360	0	0	0	0	360
Sum (mg/kg day)	1.12	112	224	504	16	36	48	109	360

*See Christiansen and coworkers (4) for estimates of high-end human intakes and for the adjusted intakes used as basis for the mixture study. ^a^Dams were dosed with paracetamol alone or in mixture only from GD 13 to 19 and after birth from PD 14 to 22, in order to avoid problems with parturition. For more information on the rationale behind this, see Axelstad and coworkers (5).

Mixtures and vehicle were administered to the dams by oral gavage from GD 7 to the day before expected birth (GD 21) and again from the day after birth, PD 1–22. All doses were given in vehicle (2 mL/kg) via oral gavage ((5) for details on dosing regimen). The dams were inspected twice a day for general toxicity and body weights were recorded on GD 4 and daily during the dosing period to monitor weight gain and to adjust dose according to weight.

### *In vivo* measurements

At PD 22, one to two males per litter were weaned. From PD 39 to 50, onset of puberty was registered daily in all weaned male offspring and assessed at the time of balano-preputial separation. Age and body weight were recorded on the day that balano-preputial separation was first observed.

### Necropsies

At PD 16 and 22, approximately one male pup per litter was killed by decapitation, body weights were determined and testes were excised, weighed and prepared for histological examination.

The weaned males (approximately one to two per litter) were killed by decapitation after CO_2_/O_2_ anesthesia at 10 months of age (PD 300) and body weight was determined. Weights of levator ani/bulbocavernosus muscle (LABC), both testes, and one epididymis (alternately left and right) were measured, and sperm count analysis was performed using computer-assisted sperm analysis (CASA). For this analysis, alternately left or right cauda epididymis, including 1 cm of ductus deferens, was frozen in liquid nitrogen and stored at −80°C for later analysis. The cauda epididymis was thawed, weighed and prepared as described by Jarfelt and coworkers (9), and samples were analyzed using a 10× UV fluorescent objective and IDENT OPTIONS on the CASA. Ten fields were analyzed for each sample, and three counts were performed for each suspension. Counts were averaged, and data are presented as number of sperm per gram cauda.

Testes were fixed in Bouin’s. Pituitaries were removed from the skull after fixation in formalin. Testes and pituitaries were processed for paraffin embedding, sectioned and stained with H&E for histological examination.

### Histological examination

At PD 16, 22 and 300, one section per testis from the control group, the PM group and each high group mixture group (TotalMix450, AAMix450 and EMix450) were stained with H&E. In testes from PD 22 males, lumen formation and seminiferous tubular diameter was examined in four randomly selected fields per section at 10× magnification (100–200 tubules per testis). At PD 300, a detailed qualitative examination of the testes was performed taking into account the tubular stages of the spermatogenic cycle to identify effects such as missing germ cell layers or types, retained spermatids, multinucleate or apoptotic germ cells and sloughing of spermatogenic cells into the lumen. One section of pituitary gland from each male offspring was examined histologically with focus on presence of nodular hyperplasia and adenoma in pars distalis (10).

### Statistics

For all analyses, the litter was the statistical unit and alpha was 0.05. All data were assumed to be continuous and examined for normal distribution and homogeneity of variance and then analyzed by analysis of variance (ANOVA). Body weight was included as a covariate in data analyses when considered as relevant (e.g. organ weights). When more than one pup from each litter was examined, statistical analyses were adjusted using litter as an independent, random and nested factor. Each mixture (TotalMix, AAMix, EMix) and paracetamol was evaluated separately, the global Type I error was controlled by the two-tailed Holm–Šídák method for comparisons to the controls using GraphPad Prism. Morphometrical data on tubule diameter were analyzed using *t*-test as only one dose group from each mixture was analyzed. Dichotomous histological data were evaluated using the Fisher’s Exact Test in the SAS software program (SAS Enterprise Guide 4.3).

## Results

### Reproductive organ weight and testis histology PD 16 and 22

Testes weights showed statistically significant increases on PD 22 in the high doses of mixtures of all endocrine disrupters (TotalMix), the anti-androgens (AAMix) and paracetamol (360 mg/kg bw/day) (*P* < 0.05), compared to controls (Table 2), whereas no changes were seen at PD 16 (5). Tubular atrophy or dysgenetic seminiferous tubules were not observed at either PD 16 or 22. The histomorphometrical evaluation at PD 16 revealed a reduced percentage of lumen formation in seminiferous tubules of males from the TotalMix450 group (Table 3). This may indicate delayed testis development, but no differences in tubule diameter were seen, and all animals showed leptotene/zygotene spermatocytes in at least 50% of seminiferous tubules indicating normal progression of spermatogenesis. The increased testis weight at PD 22 was not related to changes in lumen formation or seminiferous tubule diameter, as no differences between dose groups were observed. There were no differences in age or body weight at sexual maturation (data not shown).

**Table 2:** Body and organ weight on PD 22 and PD 300.

	**PD22**			**PD300**					
**Dose groups**	*N*	Body weight (g)	Pooled testes (mg)	*N*	Body weight (g)	Pooled testes (mg)	Epididy-mides (mg)	LABC (mg)	Pituitary gland (mg)
Control	12	48.7 ± 2.13	0.244 ± 0.008	18	517.7 ± 10.7	4.13 ± 0.085	0.696 ± 0.0185	1.287 ± 0.0567	0.0105 ± 0.00028
TotalMix100	14	45.1 ± 1.15	0.243 ± 0.007	17	514.8 ± 13.70	4.02 ± 0.068	0.678 ± 0.0130	1.223 ± 0.0509	0.0102 ± 0.00024
TotalMix200	13	50.5 ± 1.54	0.270 ± 0.010	12	511.8 ± 16.9	4.00 ± 0.139	0.679 ± 0.0148	1.284 ± 0.0510	0.0101 ± 0.00021
TotalMix450	11	48.5 ± 1.03	0.273^a^ ± 0.007	14	493.8 ± 10.3	4.09 ± 0.100	0.670 ± 0.0299	1.326 ± 0.0592	0.0102 ± 0.00037
AAMix200	12	48.2 ± 2.04	0.256 ± 0.011	13	502.2 ± 8.1	3.86 ± 0.121	0.647 ± 0.0227	1.268 ± 0.0285	0.0104 ± 0.00034
AAMix450	10	48.0 ± 1.03	0.269^a^ ± 0.007	15	494.2 ± 10.2	4.22 ± 0.097	0.706 ± 0.0198	1.269 ± 0.0558	0.0106 ± 0.00011
EMix200	14	47.4 ± 1.82	0.240 ± 0.011	15	496.8 ± 10.6	4.14 ± 0.088	0.647 ± 0.0013	1.224 ± 0.0623	0.0102 ± 0.00023
EMix450	12	47.6 ± 0.92	0.247 ± 0.007	16	509.6 ± 8.2	4.12 ± 0.085	0.671 ± 0.0249	1.266 ± 0.0385	0.0102 ± 0.00033
Paracetamol	9	47.3 ± 1.24	0.257^a^ ± 0.007	13	507.8 ± 13.4	3.89 ± 0.094	0.657 ± 0.0180	1.335 ± 0.0540	0.0103 ± 0.00033

Data shown are means ± s.e.m.

^a^Values statistically significantly different from controls are marked in bold (*P* < 0.05).

**Table 3:** Testicular histopathology in rat offspring exposed perinatally to mixtures of endocrine disrupters (selected data).

	**PD 16**			**PD 22**			**PD 300**						
**Dose groups**	*N*	Percentage of tubules with lumen, mean ± s.d.	Tubular diameter, mean ± s.d.	*N*	Percentage of tubules with lumen, mean ± s.d.	Tubular diameter, mean ± s.d.	*N*	Normal epithelium	Seminiferous epithelium atrophy, Sertoli cell only	Seminiferous epithelium atrophy, spermatogenesis until pachytene stage	Focal atrophy 5–25% of tubules	Edema	Dilatation
Control	14	33.2 ± 27.8	82.1 ± 3.5	12	81.6 ± 8.9	111.7 ± 4.8	18	17	0	1	0	1	0
TotalMix450	10	10.5 ± 11.6*	79.7 ± 4.1	10	81.9 ± 3.7	110.9 ± 6.1	16	15	1	0	0	1	1
AAMix450	11	30.5 ± 15.3	83.2 ± 3.3	10	77.5 ± 9.9	111.4 ± 4.6	16	16	0	0	0	1	1
EMix450	10	30.8 ± 37.7	80.7 ± 4.6	9	78.0 ± 7.6	112.0 ± 3.5	16	14	1	0	1	1	1
PM	11	18.8 ± 14.3	83.5 ± 5.3	9	87.2 ± 4.5	115.6 ± 4.4	14	13	0	0	1	2	0

*Indicates value statistically significantly different from controls (*P* < 0.05, non-parametric Dunn’s test).

### Sperm count PD 300

**Figure 1:**
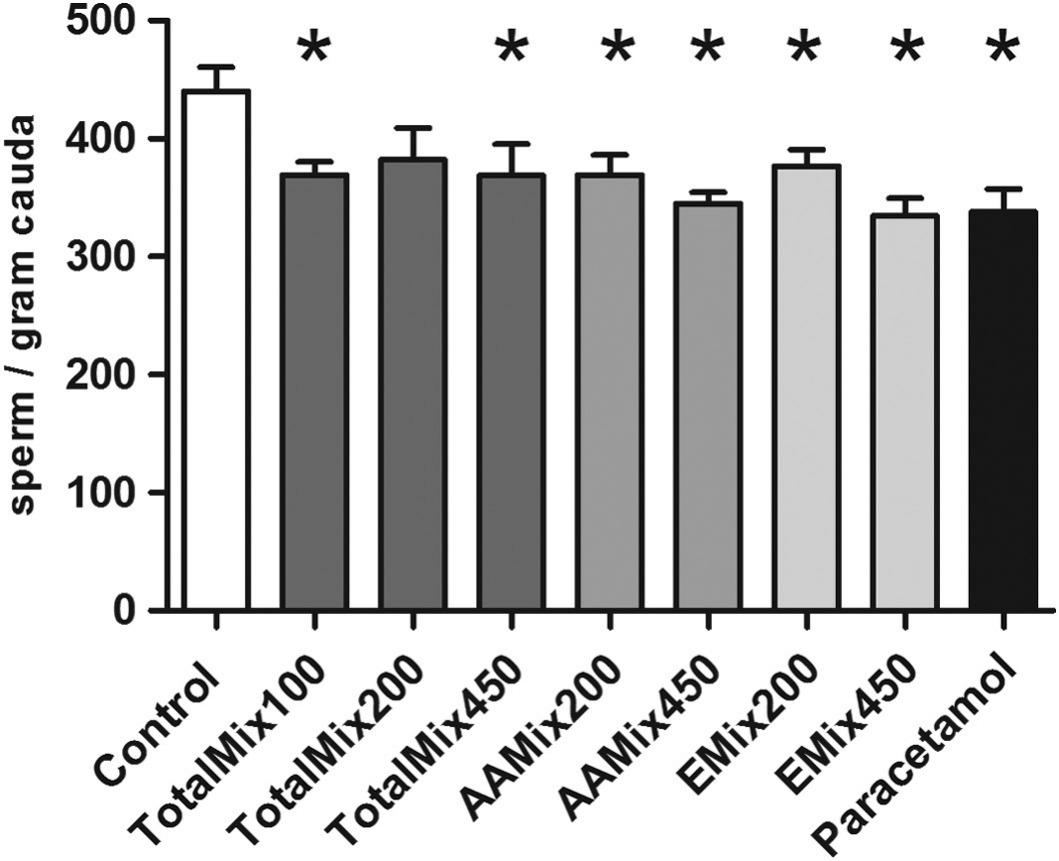
Number of sperm per g cauda epididymis in 300-day-old male rats (*N* = 14–20). Group means and s.e.m. are shown. Statistically significant fewer sperm per g cauda was observed in all except one dose group compared to control. Analysis of variance (ANOVA) test and Holm–Sidak correction was used *P < 0.05. The control value in this study is not significantly different from historical control values of epididymal sperm counts in adult male Wistar rats from our previous studies (515 ± 23, 521 ± 46, 364 ± 19, 490 ± 16, and 409 ± 18) (sperm/g, control mean ± s.e.m., references 8, 11, 12, 34 and 36).

Epididymal sperm counts were significantly reduced compared to controls in almost all dose groups. With the mixtures composed of anti-androgens (AAMix) and estrogenic chemicals (EMix), there was a dose-dependent trend, as sperm counts were more strongly compromised at the higher doses. The exception from these observations was that in the TotalMix-200 group, which contained one high outlier, the reduction in sperm count was not statistically significant (Fig. 1). Similar to the presented data for sperm per gram cauda epididymis, sperm concentrations not corrected for epididymis weight showed significant reductions in all dose groups except for TotalMix-200 (*P* = 0.06) (data not shown). Comparison with historical control data show no difference between controls from this study and controls from five other studies carried out in the same lab within the same period.

In this analysis, five males with tubular degeneration were omitted (see section on histology below), and only animals with apparently normal testicular histology were compared. Of the five excluded animals, four had markedly reduced sperm count (68–196 sperm/g cauda), and for one animal, sperm count could not be assessed as the epididymis was lost at dissection. As both testes and epididymides were noted as being small/atrophic at dissection, it is likely that this animal had a low sperm count.

### Reproductive organ weight and histology PD 300

At PD 300, no statistically significant effects were seen on weights of testes (pooled left and right), epididymis, levator ani/bulbocavernosus muscle (LABC) or pituitary gland (Table 2). In five animals from different dose groups, the histological evaluation of testes revealed severe atrophy affecting all seminiferous tubules. Of these, one male from the control group had general spermatid depletion with presence of spermatocytes until pachytene stage (in 100% of tubules), whereas degeneration with Sertoli cells only (in 100% of tubules) was seen in one male per group from the TotalMix200, the TotalMix450, the AAMix200 and the EMix450 groups (Table 2). Focal tubular atrophy affecting 5–10% of the epithelium was seen in three males from different groups (TotalMix200, EMix450 and PM). Mild interstitial edema and dilatation of tubular lumens were seen in a few animals. Given the distribution of findings in all dose groups, none of these findings were attributed to exposures. No significant effect on pituitary histology was observed (data not shown).

## Discussion

In recent years, trends to decreasing human sperm counts have been reported in several countries, and a concern for environmental causes has been raised. In this rat study, we found changes in adult sperm production following perinatal exposure to a common medical drug, paracetamol and to mixtures of human relevant environmental chemicals.

Sperm counts can be affected by chemicals with very diverse modes of action, and this was confirmed in this study showing reduced sperm counts with exposure to paracetamol, the AAMix of anti-androgenic compounds, the EMix of estrogenic compounds and the TotalMix. These findings confirmed our previous study in aging rats (1½ years old) exposed to the TotalMix perinatally (8). In the most affected dose groups (high-dose mixture groups and PM group), the sperm count values were 17–23% lower than those seen in the control animals. The significant findings in the present study were not due to abnormally high control values, as the mean sperm count value in the control group from the present study was in the lower range of historical controls from our laboratory (8, 11, 12, 34, 36).

Several of the chemicals present in the tested mixture have previously been shown to decrease epididymal sperm count or daily sperm production in rats, as discussed below.

Effects of paracetamol on sperm count after perinatal exposure have not previously been described, but paracetamol has been shown to act as an anti-androgen as it reduces AGD and testosterone production in fetal rats (13, 14) and impairs testosterone production in human fetal testes in a xenograft model (15). This may be related to impaired steroidogenesis, as also observed in the H295R *in vitro* assay (16). In this study, developmental exposure to 360 mg/kg bw/day of paracetamol had adverse effects on male sexual differentiation, as low adult sperm counts and changes in early testis development were seen. This dose resembles human maximally recommended daily dose of around 60 mg/kg bw/day, when considering the higher metabolic rate of rats compared to humans (4, 13). These findings emphasize a serious concern for adverse health effects in children after maternal paracetamol intake at a critical period in pregnancy.

A continuous breeding study in mice showed abnormalities of epididymal sperm, but no effects on epididymal sperm count and motility or on testis and epididymis histology in male offspring (F1) at a dietary dose of 1430 mg/kg bw/day of paracetamol (17). Several studies have examined the effects of paracetamol in adult rodents, some indicating toxicity to male reproductive organs. Oral exposure to 1000 mg/kg bw/day for 30 days impaired male fertility and sperm count markedly in adult rats, and this was associated with increased apoptosis of pachytene spermatocytes and early spermatids as well as a reduction in testicular weight and interstitial volume (18). In adult mice, intraperitoneal exposure to 400 mg/kg bw/day for 5 days caused delayed spermatogenesis (19). Together with the previously published adverse findings in males rat exposed perinatally to paracetamol, i.e. increased NR, decreased LABC weight on PD 16 (5), histological changes in male mammary gland tissue on PD 55 (7) and altered gene expression in the early postnatal brain (20), these effects on prepubertal testes and adult sperm count may cause concern for the use of paracetamol in pregnancy, especially during the critical windows of fetal sexual development.

Among the chemicals in the anti-androgen mixture, several are known to be able to affect sperm quality, though mainly at higher doses than included in this mixture. For many of the investigated anti-androgens, no observed adverse effect levels (NOAELs) for adverse effects on semen parameters have not been established, but in previous studies, effects of the single compounds have often been present around 100 mg/kg bw/day or higher.

In the AAmix200 – the lowest AAmix affecting sperm count – the phthalates DEHP and DBP were present at doses of 4 and 2 mg/kg bw/day, respectively. These doses are lower than NOAELs in comparable studies with developmental exposure to single compounds. For DBP, decreased sperm count was seen at 100 mg/kg bw/day (21), but not at 50 mg/kg bw/day (22). In one study, DEHP doses of 15 mg/kg bw/day and above reduced daily sperm production in offspring (23), while other studies pointed to higher NOAELs with effects at 500–1000 mg/kg DEHP (24, 25) or no effects (9, 26). The anti-androgenic pesticides and pesticide metabolites linuron, vinclozolin, procymidone, prochloraz, pp′DDE and epoxiconazol were included in the AAMix200 group at dose levels of between 0.12 and 3 mg/kg bw/day (Table 1). For these compounds, no studies have shown effects on sperm count in this low-dose range, and mainly, higher doses have been studied. Adverse effects on semen quality parameters have been reported at doses between 50–100 mg/kg bw/day for procymidone (27), pp′DDE (28, 29) and vinclozolin (30, 31). For prochloraz and epoxiconazol, no effects on sperm parameters were seen at 10–30 mg/kg bw/day (32, 33). Collectively, the reduced sperm count in both AAMix groups is likely due to additive effects of these compounds, as effects were seen at mixed exposure to chemical doses that singly would not result in such effects.

Exposure to the 4 predominantly estrogenic compounds had a similar effect on sperm counts as exposure to the anti-androgenic compounds. In the EMix 200 group – the lowest EMix dose affecting sperm count – the butylparaben dose was 12 mg/kg bw/day. It is quite plausible that this compound is responsible for the effect of the EMix on sperm count, as our recent study showed a 23% decrease in sperm count in adult male rat offspring at 10 mg/kg bw/day and above (34). This finding supported previous studies reporting reduced sperm count after developmental exposure of rats to 100 and 200 mg/kg bw/day (35). For the UV-filter 4-MBC, no data on effects on semen quality could be located in the open literature, whereas developmental exposure to OMC showed decreased sperm counts at doses of 500 mg/kg bw/day and above (36, 37), whereas no NOAEL for this endpoint has been determined. In the EMix200 group, a BPA dose of 0.3 mg/kg bw/day was used. No previous studies have shown effects on sperm counts around this dose, although some studies have shown effects at either lower or higher doses (12, 38, 39, 40, 41, 42, 43).

For each of the mixtures AAMix and EMix, the mean responses seemed to increase with dose, and the same was seen for the TotalMix medians (data not shown). Interestingly, the effects did not appear to increase in severity after combined exposure to all three classes of chemicals, as the observed decreases in sperm counts were not greater in the TotalMix groups compared to the effects observed in the corresponding doses of AAMix, EMix or paracetamol. It appears that there is a limit to the magnitude of effect of perinatal exposure to these mixtures, and shallow dose–response curves for sperm count have also been seen with other compounds. For example, perinatal exposure to butylparaben caused 22–24% reduction in epididymal sperm count at doses from 10 to 500 mg/kg bw/day (6).

This late-life effect on sperm count was predicted by early changes in AGD, nipple retention and reproductive organ weights seen for the TotalMix, AAMix and paracetamol (Supplementary Table 1). Additionally, early testicular development was examined and effects were seen with exposure to anti-androgenic compounds (TotalMix 450, AAMix450), but not with estrogens. It may be speculated that delayed lumen formation in anti-androgen-exposed animals at PD 16 may point to delayed Sertoli cell maturation, and that this in turn leads to increased testis weight at PD 22 due to continued proliferation of Sertoli cells. However, that would be expected to persist into adulthood, which was not the case in the present study. Altered Sertoli cell maturation was described in rat offspring perinatally exposed to the thyrotoxic compound propylthiouracil (PTU), but in contrast to the current study, increased testis weights in PTU-exposed animals persist into adulthood (44), and sperm counts increase (45). Other mechanisms including changes in fluid dynamics may be included, and our observations warrant further studies on how early endocrine-mediated changes in testicular development may have late-life consequences, and how chemicals with different effect patterns in prepuberty can induce similar late-life reductions of sperm count.

Sperm count appears to be the most sensitive investigated endocrine-sensitive endpoints in males as it is affected at the lowest dose of the TotalMix (Fig. 1). Samples for investigation of sperm motility or morphology, which might have provided further valuable knowledge regarding adverse effects on male reproductive function, were not available. Also female gonads appeared sensitive to this mixture, as signs of early reproductive senescence were seen at the lowest TotalMix dose, though these findings were not as consistent across groups as the sperm count effect (46). At the next dose levels (the TotalMix-200 and -450 and the AAMix-200 and -450 groups), a few more endpoints were affected including increased nipple retention, decreased AGD and reduced prepubertal ventral prostate weight (5) (Supplementary Table 1). In adulthood, histological changes were seen in ventral prostate and male mammary gland at the high dose (6, 7, 8). Some of these effects persisted into senescence as epididymis and ventral prostate were seen together with an increased frequency of pituitary adenomas in the AAMix450 group when following offspring until 1½ years of age (8).

A safety margin of 100 between human exposures and NOAELs in rodent studies is generally considered necessary for chemical risk assessment. As the TotalMix-100 could not be considered a NOAEL, but a LOAEL, the safety margin appears to be below 100 for this mixture containing 100-fold high-end human exposure doses and paracetamol doses 7-fold human maximally recommended doses. When evaluating the effects of mixtures without paracetamol, 200-fold ‘high-end human exposures’ were able to reduce sperm count, and no NOAEL could be obtained. Thus, these findings identify a concern that pregnant women today are not sufficiently protected against endocrine-disrupting effects of chemical exposures.

## Conclusion

The present study showed that developmental exposure of rats to paracetamol and to human relevant mixtures of EDCs induced long-lasting adverse effects on the male reproductive system. Epididymal sperm counts in adulthood were significantly decreased in all exposure groups despite diverse modes of action. As effects were evident in all exposure groups, these findings cause concern that human exposure to paracetamol, environmental anti-androgens as well as environmental estrogens may contribute to the low semen quality seen in many human populations today. No NOAELs have been determined for paracetamol or for these mixtures, but the present findings point out that for anti-androgens, significant reductions in sperm numbers can occur after mixture exposure to doses of chemicals that singly would not be expected to result in any adverse effect. As the safety margin from the effective doses in these rodent studies is below 100 compared to high-end human exposures to these chemicals, this causes concern that pregnant women today are not sufficiently protected against endocrine-disrupting effects of chemical exposures.

### Supplementary Material

Supporting Table 1

### Declaration of interest

The authors declare that there is no conflict of interest that could be perceived as prejudicing the impartiality of the research reported.

### Funding

This work was funded by a grant from the European Commission, 7th Framework Programme, CONTAMED (Contaminant mixtures and human reproductive health – novel strategies for health impact and risk assessment of endocrine disrupters), grant agreement no: 212502) and by funding from the Danish Environmental Protection Agency.
