# Supplementary material for: EDC IMPACT: Reduced sperm counts in rats exposed to human relevant mixtures of endocrine disrupters
Source: Endocr Connect. 2017 Nov 28;7(1):139–48. doi: 10.1530/EC-17-0307 (PMC5776667; doi:10.1530/EC-17-0307)
Supplement: Supporting Table 1 [file ec-7-139-t001.pdf]

Supplementary Table S1. Adverse effects on reproductive development after exposure to human relevant mixtures of endocrine disrupting chemicals.

	Neonatal/prepubertal offspring						Adult offspring				
	Decreased male anogenital distance PD 1	Increased nipple retention PD 14	Decreased ventral prostate weight PD 16 or 22	Decreased LABC weight PD 16	Increased testis weight PD 22	Increased mammary gland outgrowth in females PD 22	Mammary morphology PD 55 or 300	Prostate histology PD 55 or 300 or 550	Decreased sperm count PD 300 or 550	Signs of early reproductive senescence in females PD 300 or 550	Pituitary adenoma PD 550
TotalMix100	-	-	-	-	-			-	* (g)	* (f)	
TotalMix 150	-	* (a)	-	-	-			-	* (b)	-	-
TotalMix200	* (c)	-	-	-	-				-	* (f)	
TotalMix450	* (c)	* (a, c)	* (a,c,d)	-	* (g)	* (e)	* (e)	* (d)	* (b,g)	* (f)	-
AAMix200	* (c)	* (c)	* (c,d)	-	-				* (g)	* (f)	
AAMix 450	* (c)	* (a, c)	* (c,d)	* (c)	* (g)	-	* (e)	* (b,d)	* (b,g)	* (b,f)	* (b)
EMix200	-	-	-	-	-	* (e)			* (g)	-	
EMix450	-	-	-	-	-	* (e)	* (e)	-	* (g)	-	
Paracetamol	-	* (c)	-	* (c)	* (g)	-	* (e)	-	* (g)	* (f)	

Mixture abbreviations and description of mixture composition and dose levels are presented in Table 1. For each endpoint, adverse effects are summarized, followed by a reference to the paper where these findings are described in more detail.

*** designates a statistically significant effect on this endpoint at the given dose level, and – designates lack of effect on this endpoint. Gray boxes represent endpoints not investigated at the given dose level. (a) Christiansen et al. 2012, (b) Isling et al. 2014, (c) Axelstad et al. 2014, (d) Boberg et al. 2014, (e) Mandrup et al. 2014, (f) Johansson et al. 2016, (g) current paper.**
